# Practice facilitation to promote evidence-based screening and management of unhealthy alcohol use in primary care: a practice-level randomized controlled trial

**DOI:** 10.1186/s12875-020-01147-4

**Published:** 2020-05-20

**Authors:** Alison N. Huffstetler, Anton J. Kuzel, Roy T. Sabo, Alicia Richards, E. Marshall Brooks, Paulette Lail Kashiri, Gabriela Villalobos, Albert J. Arias, Dace Svikis, Beth A. Bortz, Ashley Edwards, John Epling, Deborah J. Cohen, Michael L. Parchman, Jonathan Winter, Patricia Wessler, Timothy J. Yu, Alex H. Krist

**Affiliations:** 1 Department of Family Medicine and Population Health, Virginia Commonwealth University, One Capitol Square, Room 637, 830 East Main Street, Richmond, VA 23219 USA; 2 Department of Biostatistics, Virginia Commonwealth University, Richmond, VA USA; 3 Department of Psychiatry, Virginia Commonwealth University, Richmond, VA USA; 4 Department of Psychology, Virginia Commonwealth University, Richmond, VA USA; 5 Virginia Center for Health Innovation, Henrico, VA USA; 6 Department of Family and Community Medicine, Virginia Tech Carilion School of Medicine, Roanoke, VA USA; 7 Department of Family Medicine, Oregon Health & Science University, Portland, OR USA; 8 MacColl Center, Kaiser Permanente of Washington Health Research Institute, Seattle, WA USA; 9 Shenandoah Valley Family Practice Residency, Virginia Commonwealth University, Front Royal, VA USA; 10 Riverside Family Medicine Residency, Virginia Commonwealth University, Newport News, VA USA; 11 St. Francis Family Medicine Residency, Virginia Commonwealth University, Midlothian, VA USA

**Keywords:** Unhealthy alcohol use, Preventive care, Risk reduction, Primary care, SBIRT

## Abstract

**Background:**

Unhealthy alcohol use is the third leading cause of preventable death in the United States. Evidence demonstrates that screening for unhealthy alcohol use and providing persons engaged in risky drinking with brief behavioral and counseling interventions improves health outcomes, collectively termed screening and brief interventions. Medication assisted therapy (MAT) is another effective method for treatment of moderate or severe alcohol use disorder. Yet, primary care clinicians are not regularly screening for or treating unhealthy alcohol use.

**Methods and analysis:**

We are initiating a clinic-level randomized controlled trial aimed to evaluate how primary care clinicians can impact unhealthy alcohol use through screening, counseling, and MAT. One hundred and 25 primary care practices in the Virginia Ambulatory Care Outcomes Research Network (ACORN) will be engaged; each will receive practice facilitation to promote screening, counseling, and MAT either at the beginning of the trial or at a 6-month control period start date. For each practice, the intervention includes provision of a practice facilitator, learning collaboratives with three practice champions, and clinic-wide information sessions. Clinics will be enrolled for 6–12 months. After completion of the intervention, we will conduct a mixed methods analysis to identify changes in screening rates, increase in provision of brief counseling and interventions as well as MAT, and the reduction of alcohol intake for patients after practices receive practice facilitation.

**Discussion:**

This study offers a systematic process for dissemination and implementation of the evidence-based practice of screening, counseling, and treatment for unhealthy alcohol use. Practices will be asked to implement a process for screening, counseling, and treatment based on their practice characteristics, patient population, and workflow. We propose practice facilitation as a robust and feasible intervention to assist in making changes within the practice. We believe that the process can be replicated and used in a broad range of clinical settings; we anticipate this will be supported by our evaluation of this approach.

**Trial registration:**

ClinicalTrials.gov, ClinicalTrials.gov Identifier: NCT04248023, Registered 5 February 2020.

## Background

Unhealthy alcohol use is the third leading cause of preventable death in the US. Nearly 88,000 alcohol-attributable deaths from accidents and chronic disease occurred annually between 2006 to 2010 [1], representing 2.5 million years of potential life lost. Unhealthy alcohol use is the eighth leading cause of death and fourth leading cause of decreased quality of life [2]. It causes health problems (such as liver disease, neurologic damage, cardiovascular disease, as well as several forms of cancer) [3–6], social problems (such as depression, intimate partner violence, and child neglect) [7, 8], and economic difficulties. Excessive alcohol use costs the US $249 billion annually [9], though the real human cost of pain and suffering are not included in these figures. Despite these risks, unhealthy alcohol use is common and increasing in adults [10]. The prevalence of alcohol use disorder (AUD) has increased from 8.5 to 12.7% in the past decade. Women, blacks, and older adults had the greatest increases, by 59.8, 55.8%, and 61.9–75.0%, respectively [11]. Compounding these increases, 26.2% of adults reported binge drinking in the previous month [12].

### Defining unhealthy alcohol use

Defining unhealthy levels of drinking is difficult, with some ambiguity about drinking risk thresholds. Defining “low risk” and “high risk” drinking involves nuanced interpretation of results from epidemiological studies showing dose-response curves in relation to adverse outcomes [13, 14]. As a result, there is no consensus in defining risky drinking [13, 15]. Clinically, unhealthy alcohol use spans a wide range of behaviors, from risky drinking (i.e., drinking above recommended limits) to severe alcohol use disorder. Currently, the National Institute on Alcohol Abuse and Alcoholism (NIAAA) recommends that men age 18 to 64 years consume no more than four drinks per day and no more than 14 drinks per week; women any age and men ages 65 years and older should consume no more than three drinks per day and seven drinks per week [16, 17]. The Diagnostic and Statistical Manual of Mental Disorders (DSM-5) defines alcohol use disorder (AUD) as when a person experiences at least two of the 11 criteria shown in Table 1. The severity of the disorder is considered mild (2–3 symptoms), moderate (4–5 symptoms), or severe (6 or more symptoms) based on the number of criteria met. These categories align with the US Preventive Services Task Force (USPSTF) recommendation on screening and counseling for unhealthy alcohol use [20].

**Table 1:** Categories of unhealthy alcohol use, consistent with the USPSTF definitions

Term	Definition
Risky drinking [18]	Consumption of alcohol above recommended daily, weekly, or per occasion amounts, but not meeting criteria for alcohol use disorder. For women no more than 3 drinks per day and no more than 7 drinks per week. For men no more than 4 drinks per day and no more than 14 drinks per week. Adolescents, women who are pregnant or trying to get pregnant, and adults planning to drive a vehicle or operate machinery should avoid alcohol completely.
Binge drinking [18]	An occasion of drinking that brings blood alcohol concentration levels to 0.08 g/dL. This typically corresponds to 4 drinks for women and 5 drinks for men over 2 h.
Alcohol use disorder [19]	Pattern of alcohol use leading to impairment or distress, as manifested by two (or more) of the following in a 12-month period: **(1)** Having times when the patient drank more, or longer, than intended. **(2)** More than once wanted to cut down or stop, tried it, but could not. **(3)** Spending a lot of time drinking or being sick/getting over the aftereffects of drinking. **(4)** Wanting to drink so badly that they could not think of anything else. **(5)** Found that drinking (or being sick from drinking) often interfered with taking care of home or family responsibilities, caused problems at work, or caused problems at school. **(6)** Continuing to drink even though it was causing trouble with family and friends. **(7)** Given up or cut back on activities that were important or interesting in order to drink. **(8)** More than once gotten into situations while or after drinking that increased the chances of getting hurt (e.g., driving, swimming, unsafe sexual behavior). **(9)** Continued to drink even though it was causing depression or anxiety, other health problems, or causing memory blackouts. **(10)** Having to drink much more than previously in order to get the desired effect or finding that the usual number of drinks had much less effect than previously. **(11)** Experiencing the symptoms of withdrawal after the effects of alcohol were wearing off, such as trouble sleeping, shakiness, restlessness, nausea, sweating, racing heart, or seizure.

### Recommendation for screening and counseling in primary care

In 2013 and 2018, the USPSTF recommended that clinicians screen adults for unhealthy alcohol use and provide brief behavioral counseling to persons engaged in risky drinking – collectively called screening and brief intervention (SBI).8 [8, 21], The USPSTF found that brief one- to three-item screening tools such as Alcohol Use Disorders Identification Test-Consumption (AUDIT-C) and Single item Alcohol Screening Questionnaire (SASQ) had good sensitivity and specificity to identify the full spectrum of AUDs [22]. The validated screening questions can be found in Table 2.

**Table 2:** Two validated screening questionnaires for unhealthy alcohol use

Instrument	Questions	Positive screen
AUDIT- C[23]	1. How often do you have a drink containing alcohol? [Never, monthly or less, 2–4 times per month, 2–3 times per week, 4 or more times a week] 2. How many standard drinks containing alcohol do you have on atypical day? [1 or 2, 3 or 4, 5 or 6, 7 to 9, 10 or more] 3. How often do you have six or more drinks on one occasion? [Never, less than monthly, monthly, weekly, daily or almost daily]	Responses scored 0–4 Score > 8 is positive
SASQ [24]	How many times in the past year have you had 5 [for men] / 4 [for women] or more drinks in a day?	One or more occasions

The USPSTF identified 65 trials involving 34,294 patients evaluating brief behavioral counseling interventions [20]. Interventions resulted in reductions in the odds of both exceeding weekly recommended drinking limits and binge drinking at 6- to 12-months’ follow-up [22]. Epidemiologic data clearly links these reductions in alcohol use with reductions in risk for morbidity and mortality [25], suggesting that brief interventions would result in improvements in health outcomes. Behavioral counseling interventions varied in their specific components, delivery methods, duration and intensity (Table 3). Most interventions involved 1 to 2 sessions, had a median contact time of 30 min or less, and took place in primary care settings. One-third of interventions were delivered by primary care clinicians. Some interventions included a web-based component and three had group-based interventions. Personalized normative feedback sessions, in which participants were shown how their alcohol use compares to others was the most commonly reported intervention component. Other common intervention components included motivational techniques, ways to reduce drinking, drinking diaries, action plans, alcohol use prescriptions, and feedback on how an individual’s alcohol consumption was affecting their health. A few interventions included more extensive cognitive behavioral counseling; screening, brief intervention, and referral to treatment (SBIRT); or a stepped care approach where participants who did not reduce alcohol use after a brief intervention were graduated to more intensive interventions.

**Table 3:** Number of studies regarding treatment of AUD by intervention characteristics (total number of studies = 90)

Study Characteristic and Number	
Number of sessions	
•Single session	48
•Multiple sessions	39
Intensity	
•Very brief	18
•Brief	38
•Extended	31
Median contact minutes (range)	30 (1 to 600)
Web-based	27
Personalized normative feedback	55
Motivational interviewing	35
Cognitive behavioral therapy	10
Personalized health feedback	7
Stepped care	3
Primary care involved / delivered	44

Medication-assisted therapy (MAT) has also been shown to be an effective treatment for adults with moderate to severe AUD [26]. Acamprosate, naltrexone, and disulfiram have US Food and Drug Administration approval for treating AUD. A 2014 systematic review found that acamprosate and oral naltrexone reduce alcohol consumption for adults. Evidence related to injectable naltrexone was limited at the time of the evidence review. Evidence from randomized controlled trials did not support the effectiveness of disulfiram, but it may be recommended for those whom acamprosate and naltrexone are not suitable. No studies directly compared the effectiveness of acamprosate versus naltrexone [27, 28]. Most studies included a psychosocial cointervention when evaluating medication effectiveness.

The overarching purpose of this study is to broadly promote routine screening and counseling for unhealthy alcohol use. It will also assess the components of practice facilitation necessary to change care delivery.

## Methods

**Fig. 1:**
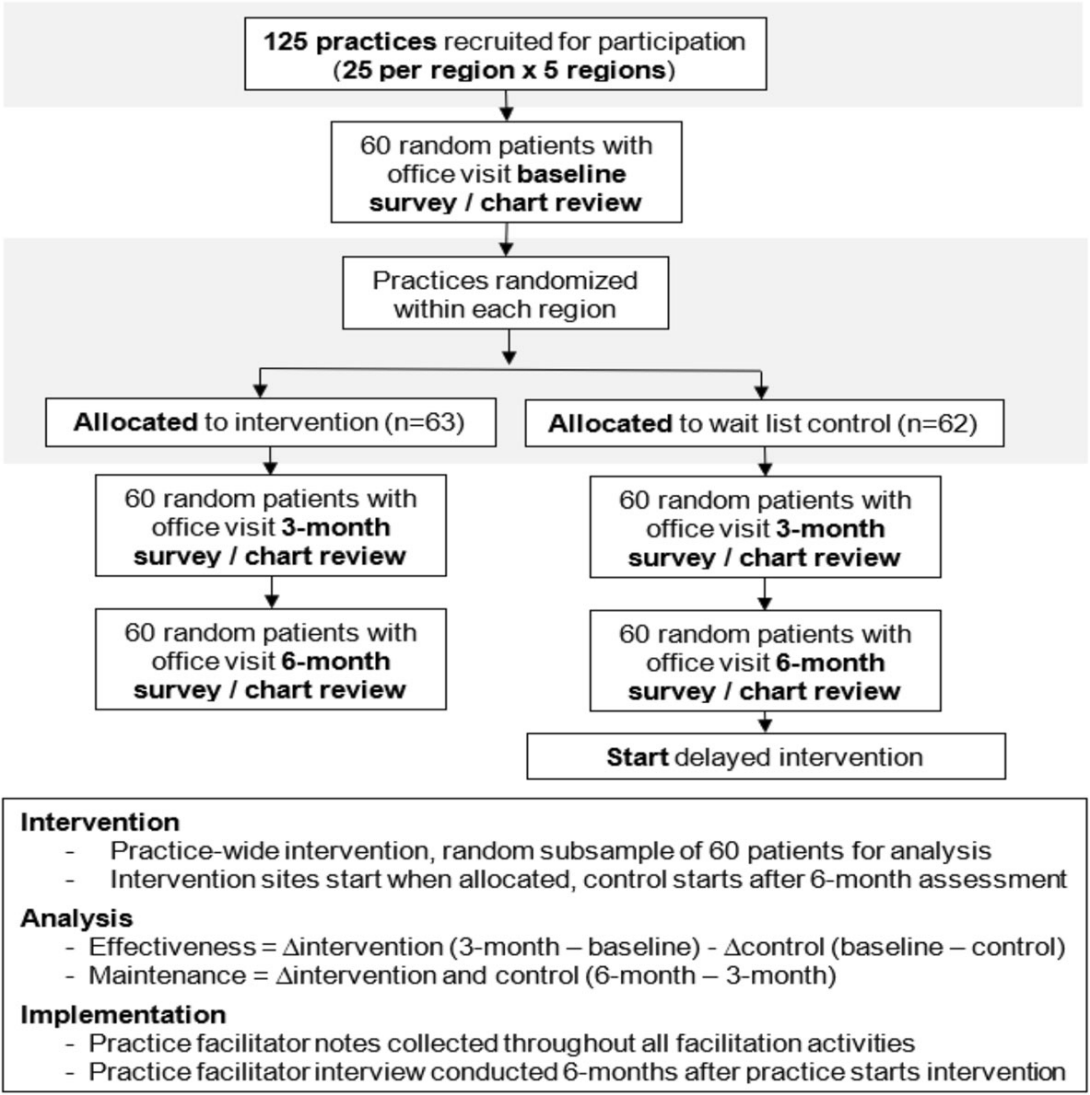
Consort – Implementation Study Flow Diagram

This is a cluster randomized trial with wait list control (Fig. 1). We will recruit 125 primary care practices distributed across five regions in Virginia, United States of America. Each region is centered around a local family medicine residency that will serve as an educational hub for practice recruitment and support. Practices will be excluded if they do not serve patients 18–65 years old. Practices will be randomly allocated in a 1:1 ratio to receive practice facilitation at startup or after 6 months delay. Practice facilitation will include provision of a facilitator, education and training, shared learning and best practices, screening and counseling toolkits, data support, and assessment with feedback. Practice activities will be locally led by a self-selected clinician, nurse, and administrator champion. The intervention will adapt and evolve over the regional rollout. Using mixed methods, we will assess the increase in screening for unhealthy alcohol use, increase in provision of brief counseling interventions and MAT, reduction in alcohol intake, and influence of practice facilitation (e.g. dose, mode, reach) and practice implementation strategies (e.g. SBI and MAT strategies and tools implemented and how implemented) on outcomes. This study has been approved by the VCU Internal Review Board, August 2019, (IRB HM20016728) and contains no more than minimal risk to participants. The risks are limited to breaches of privacy and confidentiality.

### Specific aims

#### Aim 1 (screening)

To evaluate whether practice facilitation increases screening rates for unhealthy alcohol use in primary care***.*** From patient postal survey data, chart reviews, and All Payer Claims Data (APCD), we will determine whether there is a greater increase in screening at 3 and 6 months for patients in intervention practices versus wait list control practices.

#### Hypothesis 1

Compared to control practices, 10% more patients in intervention practices will report being asked about alcohol use (increase from 78 to 86%) and 50% more patients in intervention practices than control practices will have a documented screen using AUDIT-C or SASQ (increase from about 20 to 30%).

#### Aim 2 (treatment)

To evaluate whether practice facilitation increases treatment for unhealthy alcohol use in primary care. From patient postal survey data, chart reviews, and APCD, we will determine:

*Sub-aim 2a.* whether there is a greater increase in counseling patients with risky drinking (more than 14 drinks per week for men, 7 drinks per week for women, or more than 3 drinks per occasion) at 3 and 6 months for intervention versus wait list control practices;

*Sub-aim 2b.* whether there is a greater increase in MAT for patients with moderate to severe AUD at 3 and 6 months for intervention versus wait list control practices; and.

*Sub-aim 3b.* whether patients who report risky drinking reduce the amount they drink in 6 months.

#### Hypothesis 2

Compared to control practices, 50% more patients in intervention practices with risky drinking will report or have documented treatment (increase from 20 to 30%).

#### Aim 3 (practice implementation and support moderators)

To understand the practice implementation strategies and practice support factors that influence the effectiveness of the intervention in promoting routine screening for unhealthy alcohol use. From practice facilitator field notes, facilitator interviews, and the clinician survey, we will code and qualitatively rate consolidated framework for implementation research (CFIR) constructs that influence intervention implementation effectiveness. We will specifically evaluate:

*Sub-aim 3a.* what practice strategies most benefit a practice’s ability to implement screening, counseling and treatment protocols to address unhealthy alcohol use;

*Sub-aim 3b.* what practice facilitation factors influence implementation success;

*Sub-aim 3c.* how community, organization and practice-level factors impact implementation efforts; and.

*Sub-aim 3d.* how practices adapt implementation strategies to reflect local needs and challenges.

#### Interventions and control conditions

The practices will be randomly allocated between intervention and control conditions. Allocation will be conducted by the study biostatistician, who will use the R statistical software to generate random numbers (between 0 and 1) for each clinic, allocating to the intervention for numbers greater than or equal to 0.5 and otherwise allocating to the control. Practices will not be blinded to control or intervention arm as they will be aware of their time to intervention based on enrollment.

#### Intervention condition

**Fig. 2:**
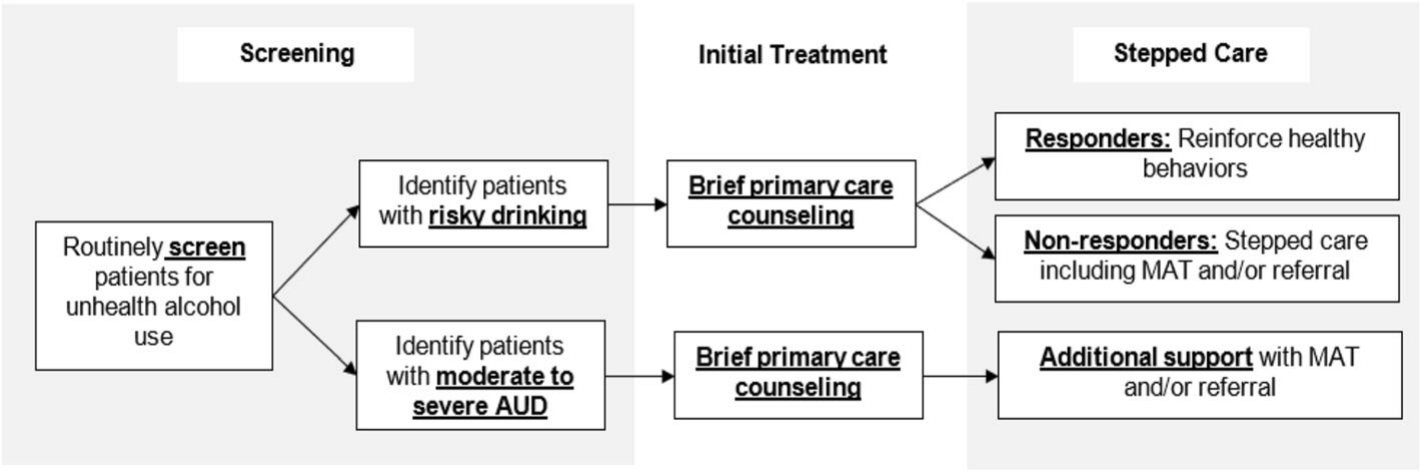
Screening, Counseling, and Treatment for Unhealthy Alcohol in Primary Care: Relationship Between SBI, Stepped Care, MAT, and Community Referral

The overall intervention is depicted in Fig. 2. This is consistent with the USPSTF recommendation, and involves systematically implementing screening, counseling, and treatment for unhealthy alcohol, including SBI, stepped care, MAT, and SBIRT.

To support intervention implementation, each practice will be asked to make seven high leverage changes:

1. Form a quality improvement team.
2. Assess practice capacity, knowledge, workflow, and needs (Table 4).
3. Attend educational sessions.
4. Commit to a screening process.
5. Commit to a counseling and treatment process.
6. Identify community referral connections.
7. Develop strategy to record care and measure performance.

**Table 4:** Initial Practice Screening and Treatment Intake Assessment

Assessment and Questions	
Screening assessment	•What is current screening practice? •Is AUDIT-C or SASQ integrated into EHR? •How do they document screening in EHR? •Can they generate screening rate measures? •Can they identify who is due to be screened? •Can they send patients screening questions through the portal? •Who would best do the screen? •When is it best to screen? •What additional supports do they need?
Treatment assessment	•What is current counseling practice? •What is current MAT practice? •What community and behavioral health supports are available? •What patient self-management material is available? •What is clinician confidence with brief counseling? •What is clinician confidence with MAT? •What help do they need with clinical-community and clinical-behavioral health connections?

#### Practice support strategies (practice facilitation)

We propose to support practices with the above activities through practice facilitation, based on the AHRQ how-to guide, “Developing and Running a Primary Care Practice Facilitation Program: A How to Guide,“ [29] the EvidenceNow practice facilitator toolkit [30], and informed by our experiences providing practice facilitation in EvidenceNow and other ACORN studies. Per the EvidenceNow revised definition of practice facilitation, our support strategies are designed to motivate, guide, and support practices in adopting, implementing, and sustaining evidence-based changes and quality improvements for unhealthy alcohol use. Our practice facilitation consists of nine key components:

1. Provision of a practice facilitator.
2. Engage leadership.
3. Financial and business support.
4. Provision of education and training.
5. Coordination of shared learning and best practices.
6. Maintenance of an online support center.
7. Creation of a change package.
8. Creation of a practice facilitator roadmap.
9. Provide assessment and feedback.

#### Control condition

Practices randomized to the control group will receive the intervention at a delayed start, 6 months after the intervention practices in their region. They will participate in data collection in parallel with intervention practices (i.e. prior to implementing changes).

#### Data collection

We propose to use nine sources to address our aims: practice facilitator field notes, clinician surveys, practice team surveys, chart review, patient surveys, Virginia All-Payers Claims Database, audio recordings, and semi-structured interviews from site visits.

#### Practice facilitator field notes

Practice facilitators will keep detailed field notes for each interaction, including who participated, type of interaction, what happened during interaction, practice progress, and decisions made. Email interactions will be saved. For planned interactions (e.g. Practice Team meetings, academic detailing sessions, screening and counseling needs intake assessment), we will develop structured templates to ensure practice facilitators record needed data elements. Field notes will be entered into RedCap using a blend of structured (e.g. attendees, type of interaction, length of interaction) and unstructured fields (e.g. challenges addressed, decisions made, perceived engagement of attendees).

#### Clinician survey

All practice clinicians will be asked to complete a survey on paper or through RedCap (whichever each clinician prefers) to assess their profile (e.g. age, gender, race/ethnicity, degree, years in practice, FTE, etc), confidence with screening and counseling for unhealthy alcohol use, understanding of the USPSTF guideline, knowledge of practice strategy for screening and counseling, attitudes towards screening and counseling, and perceived challenges and barriers to screening and counseling. The survey will be administered just prior to starting the intervention and again at the end of the maintenance period (6 months after going live).

#### Practice team survey

During the initial Practice Team meeting, the practice facilitator will guide the team to complete a survey on readiness to implement alcohol screening and the Implementation Climate Scale [31]. The readiness survey will be repeated at the end of the maintenance period. Responses will be used to help identify specific practice needs for an effective intervention implementation. Additionally, responses will be used to understand factors associated with more effective implementations.

#### Chart review

At baseline, 3 months, and 6 months, practices will be asked to perform chart reviews on 60 patients. Practice facilitators will train, coordinate, and assist practices in doing this task. Patients will be randomly selected from patients aged 18–75 seen for a chronic care or wellness office visit in the prior month. To generate the sample, practices will be asked to generate a list of all unique patients seen in the prior month. Using a random number generator, the research team will let the practice know which patients to include based on numerical ranking. Using a structured RedCap template, chart abstracters will record for each study patient: a practice assigned patient study ID, age, sex, race-ethnicity, insurance type, preferred diagnosis, active diagnoses, documented alcohol use (whether documented and what is documented), documented screening (whether screened, when screened, how screened), documented counseling (when and content of counseling), provision of MAT (when and what), referral to treatment programs (when, program, and if follow-up documented), and additional documented health behaviors.

#### Patient survey (screening-treatment)

We will mail the chart review sample of patients a postal survey. Given that there is generally poor documentation of alcohol screening and counseling, this survey will be critically important to understand screening and treatment rates and patients with undocumented risky drinking. Patient survey data will supplement chart review data to assess outcomes for aims 1 and 2 and provide contextual data for aim 3, subgroup analyses, and evaluator/AHRQ analyses. For each survey, we will optimize the response rate by using a modified-Dillman method [32–34]. Surveys will be mailed on practice stationery and in practice envelopes, including a personal note from the patient’s clinician [33]. Survey return envelopes will be addressed to the VCU research team for data entry and analysis. Surveys will include the practice assigned patient ID to link survey responses to the chart review. Only practices will be able to link the patient ID with the patient, but will not receive raw data. A cover letter will be used for patient assent, as approved by the VCU IRB. VCU will receive responses, but not be able to link responses to any patients. Surveys will ask patients basic demographic information not in the chart (education, income, marital status), whether their clinician has asked them about alcohol use in the past year, the AUDIT-C questions, whether their clinician has counseled them about healthy drinking levels or advised them to reduce the amount they drink, and whether they have been given any educational materials to help reduce alcohol use.

#### Patient survey (health outcome)

Patients who have positive AUDIT-C screen on postal survey or have documented unhealthy alcohol use on chart review will receive a follow-up survey 6 months later to reassess alcohol use, screening, counseling, and treatment. The VCU research team will send practices the list of patient IDs for the practice to resurvey using the same modified-Dillman approach. Responses will be used to calculate (i) whether risky drinkers have received subsequent screening, counseling, treatment, or follow-up, (ii) the proportion of risky drinkers who make improvements, (iii) whether risky drinkers used any educational materials or followed up on referrals, (iv) and the association between receiving primary care screening and counseling with any improvements.

#### APCD data

As part of our Medicaid expansion evaluation, we have access to statewide APCD data. The APCD includes medical and pharmacy claims submitted by commercial and public insurance carriers for over 5 million of Virginia’s 8.4 million residents [35]. All submissions include clinician National Provider Identifier number, medical professional services (diagnoses, counseling claims) and pharmacy services (MAT prescriptions). We will use this data to calculate diagnosis of unhealthy alcohol use and AUD, frequency of counseling, frequency of MAT by practice and clinician. This will augment our assessment and feedback data, outcomes assessment, and possibly serve as a long-time mechanism for monitoring practice performance.

#### Audio recordings

Practice assessment and feedback meetings and the regional learning collaboratives will be audio recorded. Recordings will be used to augment field notes.

#### Semi-structured interviews and site visits

We will identify a sub-set of eight practices from each regional cohort for participation in interviews – four high performing practices and four lower performing practices based on screening rates from the 6-month chart review (total *n* = 40). Semi-structured interviews will be conducted with each of the three Practice Team members. Interviews will assess the practice champions’ knowledge and perceptions of unhealthy alcohol use and the role of primary care in addressing it; experiences implementing SBI and MAT for unhealthy alcohol use and working with the practice facilitators; multi-level contextual factors influencing implementation, including local, organizational and health system characteristics; practices change made; uptake of changes across clinicians; and the process for making changes. Interviews will be conducted by video, digitally recorded, and transcribed.

### Analytic plan

#### Quantitative analytic plan

We will use generalized linear mixed model framework for analysis [36, 37], which will account for the nesting of patients within practices. These models will include (separately) as patient-level outcomes binary indicators of screening for alcohol use and documented evidence of screening using Audit-C or SASQ (Aim 1), treatment (Aim 2a), and MAT use (Aim 2b). Each model will include a two-level fixed group effect (intervention vs. control), and three-level fixed time effect (baseline, 3 months, 6 months), a group-by-time interaction, a patient-level random effect to account for repeated measurements, and a practice-level random effect to account for clustering of patients within practices. We will compare rates of change between intervention and control practices from baseline to 3 months, and baseline to 6 months. Adjusted comparisons will include all patient-level (age, sex, race, ethnicity, insurance type), practice-level, and implementation measures into the model as fixed effects without interaction with group, time, or the group-time interaction. Based on methods of collection, we do not anticipate dropout of practices or missing data.

#### Qualitative analytic plan

To assess our four-practice implementation and support questions (how practice implementations strategies; how practice facilitation factors; how community, organization and practice-level factors; and how adapt implementation strategies influence intervention success), we will conduct a mixed methods analysis using the CFIR framework [38–40]. Our analysis will use the semi-structured interviews from the 20 highest and 20 lowest performing practices and a more general analysis based on practice facilitator field notes for all 125 practices. Qualitative data will be managed using qualitative database software, Atlas.ti [41]. A subset of the research team will read through the full dataset several times to identify main content areas [42]. From the field notes, the team will derive the type and intensity of support provided by facilitators and reach. The team will use both template-based and emergent coding techniques to create an a priori codebook in which codes are given meaningful definitions and applied in a standardized manner for template analysis, and while coding, identify emergent codes by discovering meaningful ideas not represented in the predetermined code for emergent analysis [42, 43]. The team will follow a protocol-driven approach to analysis that includes: 1) group reading of the data to refine a priori codes, identify emergent codes, and reach agreement on code definition; 2) independent *test coding*, during which a subset of documents, selected for variation, are coded to test the operational limits of the codebook and the ability of coders to apply codes reliably and consistently; and 3) independent coding combined with scheduled merges of coded data and weekly team coding huddles for early detection of threats to inter coder reliability. Once coded, the research team will identify themes within the data [44].

### Sample size

Power calculations account for the varying treatment effectiveness between practices due to (i) practice-based randomization and (ii) nesting of patients within practice [45, 46]. Assuming a 40% non-response rate, we anticipate 25 completed patient surveys from each practice (3125 total). This provides 90% power (with 5% type-I error rate and intra-cluster correlation of 0.05) to detect (i) a 10% difference in screening rates (76% in control vs. 86% in intervention), and (ii) a 10% difference in documented screenings using AUDIT-C or SASQ (20% in control vs. 30% in intervention). Assuming a 20% AUD rate, then 5 surveys and/or chart reviews per practice (625 total) will achieve 80% power (with 5% type-I error rate and intra-cluster correlation of 0.05) to detect (i) a 10% difference in counseling rates (20% in control vs. 30% in intervention), and (ii) a 10% difference in MAT rates (20% in control vs. 30% in intervention).

### Trial status and monitoring

The timeline is shown in Table 5. This study is expected to begin enrolling practices June 2020 and continue through summer 2021. A data safety monitoring board (DSMB) – comprised of a clinical researcher, biostatistician, and research assistant – will meet annually to review findings. The DSMB will be independent and without competing interests. Patients, clinicians, health systems, and practice facilitators will be able to report adverse events to the VCU IRB and DSMB. Modifications to the existing protocol will be updated on ClinicalTrials.gov and addended with the VCU IRB.

**Table 5:** Overall Project Timeline

Study QUARTER	1	2	3	4	5	6	7	8	9	10	11	12
**Development Phase**												
IRB application	X											
Prepare practice materials	X											
Hire/train practice facilitators	X	X										
Assemble/update toolkits	X	X	X	X	X	X	X	X	X	X	X	X
**Intervention Phase (Dissemination and Implementation)**												
Region 1 activities	R	I	I	I	C	C	C					
Region 2 activities	R	R	I	I	I	C	C	C				
Region 3 activities	R	R	R	I	I	I	C	C	C			
Region 4 activities	R	R	R	R	I	I	I	C	C	C		
Region 5 activities	R	R	R	R	R	I	I	I	C	C	C	
**Data Collection and Analysis Phase**												
Regional data collection		X	X	X	X	X	X	X	X	X	X	
Midpoint and final analyses						X	X				X	X
Participation in evaluator and AHRQ collaborative activities	X	X	X	X	X	X	X	X	X	X	X	X

Note: *R* practice recruitment, *X* scheduled activity

Analysis and implications from this study will be published in medical journals and presented at medical conferences. Practices and clinicians will recieve summaries of the results prior to publication; feedback will be solicited and incorporated into manuscripts. The investigators will not have restrictions on what they can present or publish. This article presents protocol version 1.2 of the study which was finalized on July 30, 2019.

## Discussion

This study will systematically disseminate and implement evidence-based screening, counseling, and treatment recommendations for unhealthy alcohol use through a practice facilitation intervention. Practices will be asked to implement a process for routinely screening and documenting alcohol use, providing brief counseling for risky drinking, prescribing MAT for patients with AUD, and referring patients for additional support if they have moderate to severe AUD or fail brief counseling interventions. How these elements are implemented will vary based on each practices’ resources, patient population, and community programs. Given the low uptake of this preventive service and the known adverse consequences of unhealthy alcohol use, this study has great potential to improve health in Virginia. If 125 primary care practices participate, as planned, nearly 1 million Virginians will be exposed to the intervention during the study period. Long term, the study may create a cultural shift in care delivery that can further sustain and disseminate the routine screening and counseling for unhealthy alcohol use throughout primary care.

We will use a robust yet feasible practice facilitation strategy to catalyze the proposed changes. Practices will be asked to complete seven tasks to change care delivery – form a quality improvement team; assess practice capacity, knowledge, workflow, and needs; attend educational sessions; commit to a screening process; commit to a counseling and treatment process; identify community referral connections; and develop a strategy to record care and measure performance. To support these changes, we will provide nine key practice facilitation supports – provision of a practice facilitator; engage leadership; assist with strategies for financial and business support; provision of education and training; coordination of shared learning and best practices; maintenance of an online support center; creation of a change package; creation of a practice facilitator roadmap; and provide assessment and feedback.

We believe that this process of facilitating practice change can be broadly replicated across settings and for a range of care delivery needs. We will conduct a robust evaluation of the approach, including both practice factors and facilitation supports, to inform future interventions. Adding to our work, this project will occur in collaboration with five other research centers in Colorado, Illinois, Michigan, North Carolina, Oregon who are similarly providing practice facilitation [47]. Findings will be shared across all research centers to better inform screening and counseling for unhealthy alcohol use and the process of practice facilitation throughout the nation.

## Data Availability

Not applicable at this time as no data has been collected.

## Abbreviations

AHRQ: Agency for Healthcare Research and Quality
ACORN: Virginia Ambulatory Care Outcomes Research Network
APCD: All payer claims database
AUD: Alcohol use disorder
AUDIT-C: Alcohol use disorders identification test-consumption
CFIR: Consolidated framework for implementation research
DSM-5: The Diagnostic and Statistical Manual of Mental Disorders
EHR: Electronic health record
IRB: Institutional Review Board
MAT: Medication Assisted Therapy
NIAAA: National Institute on Alcohol Abuse and Alcoholism
SAS-Q: Single item alcohol screening questionnaire
SBIRT: Screening, brief intervention, referral to treatment
UAU: Unhealthy alcohol use
USPSTF: US Preventive Services Task Force
VCU: Virginia Commonwealth University
